# Polymer-Based Additive Manufacturing for Orthotic and Prosthetic Devices: Industry Outlook in Canada

**DOI:** 10.3390/polym15061506

**Published:** 2023-03-17

**Authors:** Chowdhury Sakib-Uz-Zaman, Mohammad Abu Hasan Khondoker

**Affiliations:** Industrial Systems Engineering, Faculty of Engineering and Applied Science, University of Regina, Regina, SK S4S 0A2, Canada

**Keywords:** prosthetics, orthotics, prosthesis, orthosis, additive manufacturing, 3D printing, orthotist, prosthetist

## Abstract

The conventional manufacturing methods for fabricating orthotic and prosthetic (O&P) devices have been in practice for a very long time. Recently, O&P service providers have started exploring different advanced manufacturing techniques. The objective of this paper is to perform a mini review on recent progress in the use of polymer-based additive manufacturing (AM) for O&P devices and to gather insights from the O&P professionals on the current practices and technologies and on the prospect of using AM techniques in this field. In our study, first, scientific articles on AM for O&P devices were studied. Then, twenty-two (22) interviews were conducted with O&P professionals from Canada. The primary focus was on five key areas: cost, material, design and fabrication efficiency, structural strength, functionality, and patient satisfaction. The cost of manufacturing the O&P devices using AM techniques is lower as compared to the conventional methods. O&P professionals expressed their concern over the materials and structural strength of the 3D-printed prosthetic devices. Published articles report comparable functionality and patient satisfaction for both O&P devices. AM also greatly improves design and fabrication efficiency. However, due to a lack of qualification standards for 3D printed O&P devices, 3D printing is being embraced more slowly in the O&P business than in other industries.

## 1. Introduction

### 1.1. Background

In 2017, it was reported that from 2006 to 2012, there was a total of 44,430 lower limb amputations in Canada (~7400 per year), and the average age-adjusted rate of lower limb amputation was 22.9 per 100,000 individuals [1]. Although the need is progressively increasing, the O&P industry is not still fully utilizing the emerging new technologies which have great potential to improve the quality of life of those needing orthotic and prosthetic (O&P) devices [2].

**Figure 1:**
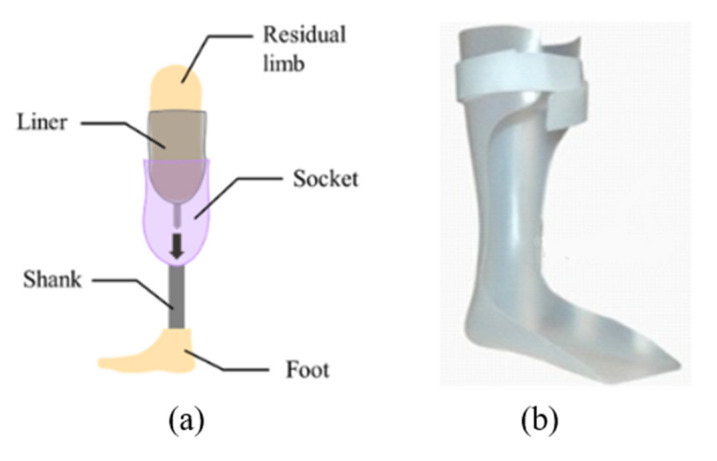
(**a**) Lower-limb prosthesis [16] © 2023 Authors. Licensee MDPI. Used under CC-BY 4.0 (**b**) Ankle-Foot Orthosis [14] © 2023 Elsevier. Used with Permission.

Prostheses are devices that restore the functionality of a missing body part, whereas orthoses are assistive devices that restore the stability and mobility of a body part of those with neuromuscular and/or musculoskeletal impairments [3]. Figure 1 represents typical prosthetic and orthotic devices. Personalized O&P devices with better fit and proper alignment are found to be crucial for users’ satisfaction when treating patients [4]. Also, providing the patient with a comfortable O&P device within 4 weeks after amputation or impairments significantly decreases the user abandonment of the devices [5]. Therefore, a rapid fabrication technique and comfortable fit of the O&P devices are two key factors that can significantly improve patients’ life. The currently practised conventional manufacturing process of O&P devices requires a significant amount of time to ensure a custom fit and involves a considerable number of manual operations and design iterations producing large amounts of waste [6,7]. However, they have been empirically tested and proven to provide patients with safe, reliable, and lasting O&P devices. Recently, many researchers as well as O&P practitioners have started evaluating advanced manufacturing technologies for producing high-performance O&P devices., Several different types of personalized foot orthoses have been created using AM technologies. These orthoses have been compared to traditionally fabricated items utilizing gait analysis and subjective assessments of fit and comfort [8,9,10,11]. These findings not only demonstrate that the AM approach to customizing foot and ankle-foot orthoses is feasible, but they also point to the significant clinical potential for this method [7]. Similarly, prosthetic sockets produced using AM techniques demonstrated enhanced comfort, increased step symmetry, and comparable lower extremity joint performance when compared to the definitive prosthetic devices [7,12,13]. However, there are still clinical, economical, and technological hurdles to overcome before AM can be applied on a large scale in a service system for the production of customized orthoses and prostheses [14,15].

The following sections will describe the conventional manufacturing processes and the latest advanced manufacturing technologies available for O&P devices.

### 1.2. Conventional Manufacturing Processes of O&P Devices

Laura Trevelin, (RTP)c (June 2022) provided a vivid description of the conventional manufacturing of prostheses which starts with wrapping the residual limb with plaster bandages and making a cast. After the cast dries, it is taken off, which serves as the negative mold, which is then filled with plaster to get the positive mold. Then, the positive mold is modified manually to adjust the amount and location of pressures on the limb. The appropriate suspension method and componentry are decided based on the patient’s need. Then the diagnostic ‘check’ socket (to check the fit) is fabricated using clear and heat moldable thermoplastics. Once it is assembled with the componentry chosen in the correct standard neutral alignment, the “check” socket is tested with a patient to evaluate its alignment and fit.

Alternatively, instead of taking the plaster cast of the residual limbs, the shape of the limb can also be captured by using a 3D scanner. Then, computer-aided design (CAD) software is used to relieve the pressure-sensitive areas and reinforce the areas where needed. Once the design is finalized, a positive model is carved out of foam e.g., expanded polystyrene (EPS), and then the check socket is made from that carved model using the steps described above.

The patient wears the check device for 4 to 6 weeks to check the fit and alignment and assess comfortability. If the fit and alignment are appropriate, a copy of the socket with an impression material (e.g., Jeltrate alginate) is taken and refilled with plaster to have a mold for the final socket. Alternatively, the check socket can be filled with plaster and cut off. If the fit is not appropriate, the check socket would be refilled with plaster, and further and more drastic changes to the shape would be made. In some cases, the suspension method could be changed altogether. Then another check socket would be created by the technician and more fittings would be required. The back and forth of the initial fitting is what takes the longest.

**Figure 2:**
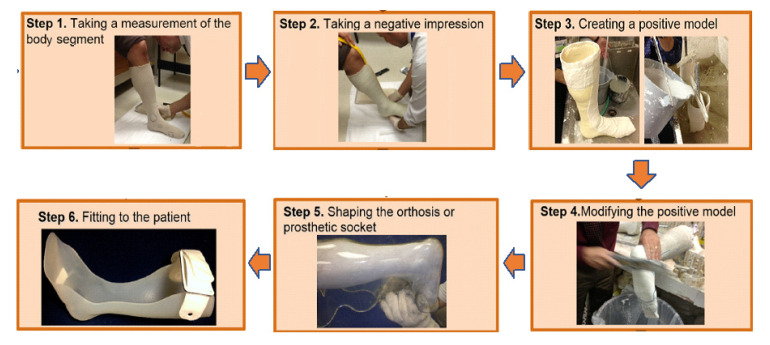
The conventional manufacturing process of O&P devices [14] © 2023 Elsevier. Used with Permission.

Once the final socket shape is tested, approved, and copied; and the final plaster mold is completely dry, it would be laminated by the technician. Using stretchable polyvinyl alcohol (PVA), bag the plaster cast is covered so that it acts as a separator. Then layers of ‘nyglass’ (a blend of nylon and fiberglass), fiberglass, and carbon fiber are added based on the empirical experience of the prosthetist that is appropriate for the patients, taking into consideration their weight, activity level, and socket suspension type. After the layers are applied, a final PVA bag funnel is put over it and the bags are sealed together with tape hooked up to a vacuum hose to draw all the air out between the layers. Acrylic resin is added and carefully coated all over the socket, taking care not to cause any wrinkles to the materials or create air pockets. The funnel is sealed off and left to be cured for 1 h. Then a cast saw is used to cut the trim lines off, break the plaster out, trim and grind the edges. The device is then reassembled onto the original setup, taking care to preserve the alignment. The patient still may need small adjustments in the alignment and fit every once in a while, especially for new amputees whose shape changes quite a bit in the first 2 years, and sometimes they need to make a whole new socket within 6 months to a year post initial fit. Figure 2 depicts the conventional manufacturing process for O&P devices.

The conventional manufacturing process of orthoses is somewhat similar to that of prostheses, with the exception that, unlike prostheses, no “check” device is made in orthoses. The procedure begins with taking measurements of the body component. Next is the molding process, in which the impression of the orthosis is taken using a casting method. After that, liquid plaster is poured into the negative mold to create a positive mold. Drying the mold might take anywhere from two to forty-eight hours. The positive mold must then be corrected and polished. Once the positive form is dried and smooth, the negative mold is removed. The model is then stapled to the Plastazote or plastaform foam, and a thermoplastic (typically, polypropylene (PP), polyethylene (PE) or other co-polymers) is pulled over it [17].

After that, the thermoplastic is heated up in the oven. The elevated temperature softens the thermoplastic material so that it may be molded over the model by the technician. The mold is detached from the model and uneven edges are smoothed off. This is the point at which the orthotic device is ready for the first fitting. Adjustments are usually required, which may be time-consuming. If significant levels of adjustments are anticipated, a completely new orthosis may be required. In such circumstances, the procedure might have to be restarted from the beginning [17].

### 1.3. Additive Manufacturing for O&P Devices

Additive Manufacturing (AM), also known as 3D printing has emerged as one of the core components of the fourth industrial revolution, Industry 4.0 [18]. In AM technology, a part is manufactured by selectively adding material in 3D volume resulting in minimal waste [19,20]. This digital but toolless manufacturing technique also allows rapid design iteration, low-cost, lightweight parts and extensive personalization which are particularly crucial for O&P devices [2]. Although clinicians’ knowledge about patient care is invaluable, to remain competitive they need to adopt advanced manufacturing [21]. Among other AM processes, Fused Deposition Modeling (FDM), Selective Laser Sintering (SLS), Material Jetting (MJ) and Vat Photopolymerization (VPP) are the four categories of AM techniques that can be used to 3D print polymer materials suitable for O&P devices [22,23]. While the direct write nature of FDM technology allows printing O&P devices without any size restrictions, the inherent anisotropy and poor inter-layer adhesion resulting from this technology often limit its suitability for safe O&P devices [24,25]. On the contrary, SLS results in isotropic material properties, however, its limited material choice often restricts the use of this AM technology in the field [26]. Many researchers and O&P practitioners have utilized AM technologies to print O&P devices and evaluated the performance of AM-processed O&P devices against the performance of conventionally manufactured O&P devices. For instance, Pousett et al. investigated the structural strength of transtibial sockets fabricated using conventional manufacturing and FDM [27].

Design for additive manufacturing (DfAM) can play a significant role in improving performance and solving problems for O&P devices. It encourages clinicians to explore new design solutions to take advantage of the unique capabilities of AM. Three advantages of using DfAM have been reported: customization, cost-effectiveness, and repeatability [28]. It has been found that DfAM improves cost-effectiveness, shortens production time, lowers wastage, and reduces human involvement when fabricating highly customized devices, compared to conventional manufacturing [29]. Interestingly, intricate designs do not affect the cost of production as the cost in AM is mainly determined by the volume of the part rather than its complexity [30]. Topologically optimized O&P devices, enabled by DfAM, were demonstrated to reduce material volume while increasing mechanical robustness [30]. The true design freedom of infill or lattice parameters, offered by DfAM, allows heterogeneous builds of O&P devices realizing a range of spatial Shore hardness is unique [31]. Finally, through DfAM the design data is captured and stored in a digital environment which permits scalable repeatability with minimal effort [28].

This article will review the findings of recently published research to assess the prospects of AM technologies in this field. Although there are some review articles published in the literature on this topic, those academic articles do not discuss the perspectives of professionals in this field on the suitability of AM for O&P devices. In this work, the professionals were interviewed to gather experts’ opinions on this topic.

## 2. Methodology

O&P professionals were contacted for one-to-one virtual interviews (a questionnaire is provided in the Supplementary Document) where they were asked to share their perspectives on the current practices and technologies used in the field of O&P, and the prospect of applying AM techniques in this field. The professionals we contacted primarily comprised certified prosthetists and certified orthotists who directly deal with the patients, registered technicians who work to manufacture the devices, and researchers who are conducting advanced research in this field. To search for the contact information of the O&P professionals, we used the database of Orthotics Prosthetics Canada (OPC), which is the representative national organization for professionals in this industry [32]. In total, we conducted twenty-two (22) interviews each lasting for around 30 min. Interviewee consent was taken before the interview and then written permission was taken to cite them in the paper. The information about the interviewees is provided in the Supplementary Document Section S1: Table S1: interview list of O&P professionals, and Section S2: interview questionnaire. Insights from interviews can be categorized into five key areas of cost, material, design and fabrication efficiency, the structural strength of the parts, and functionality and patient satisfaction. A systematic review was also performed on the journal papers published from 2010 to 2022 on 3D printing of O&P devices which addressed different issues raised by the professionals in those five areas. The literature search was conducted on ScienceDirect, PubMed, and Google Scholar from 2010 to 2022 using keywords in several variations and combinations such as “orthosis”, “prosthesis”, “3D printing”, “additive manufacturing”, “cost”, “material”, “design and fabrication efficiency”, “structural strength of the parts”, and “functionality and patient satisfaction”. English peer-reviewed papers that discussed the use of 3D printing in the fabrication of orthotic and prosthetic parts and the effect in those five key areas were selected for review.

## 3. Results and Discussion

This section summarizes the insights and feedback gathered during interviews as well as the findings from published literature.

### 3.1. Overall Manufacturing Cost

M. Prystai, (CPO)c (May 2022), S. Scott, (CP)c (May 2022), L. Trevelin, (RTP)c (June 2022) provided information on the cost of the prosthetic devices. The cost of manufacturing an upper limb prosthesis ranges anywhere from CA $6000 to $10,000. The cost of a transtibial device (below knee amputation) usually ranges between CA $4000 to $12,000, whereas that of a transfemoral device (above the knee) ranges between CA $6000 to $18,000. This apparent high cost can be attributed to the time-consuming, labour-intensive, and wasteful nature of the conventional manufacturing process.

There have been a few attempts by researchers to compare the cost of prostheses fabricated by AM method and conventional methods. Table 1 provides a comparative summary of the two processes. Day & Riley designed a partial hand prosthesis by capturing the shape using an alginate cast, converting the photographs of the cast into digital images by photogrammetry software, modifying the 3D model using Meshmixer CAD software and testing the structural integrity by finite element analysis. Then they fabricated the device using Zmorph FDM (Fused Deposition Modeling) 3D printer using Polylactic Acid (PLA) filament. Using Pugh Matrix, the patient’s satisfaction with the device was assessed. The assessment showed a high level of satisfaction in terms of fitting, comfort, function and cosmesis. The authors also reported that the cost of the 3D-printed device was approximately 56% lower than that of the laminated device [33].

De Vivo Nicoloso et al. developed a digital workflow to design and manufacture a custom-made, cost-effective, and functional 3D-printed below-knee prosthesis. To ensure the accuracy of the 3D model of the residual limb, they employed the photogrammetry technique and light scanning. For photogrammetry, they used a camera and a processing software named Metashape (Agisoft, St. Petersburg, Russia). For light scanning, they used Omega 3D Scanner (Willow Wood, Mt. Sterling, OH, USA) and its software package. The 3D model was created and modified using Fusion 360 and Meshmixer to distribute the load properly and to prevent the development of any hot spots. The pylon was designed as per the requirements of ISO standard 10328:2006 which specifies the structural requirements. The topology optimization to create an optimum structure was done using Inspire (Bristol, GB). The device was printed in an FDM printer using PLA and PETG (Polyethylene terephthalate glycol) for diagnostic purposes and PA-12 (Polyamide) for end-use purposes. The authors reported that by using the digital workflow and 3D printing technique, they were able to reduce the average cost, weight, and time of production by 95%, 55% and 95%, respectively as compared to traditional prosthetic devices [34]. It should also be noted that this reduction of cost is mainly for the fabrication of the socket, other components are still needed to be connected to the socket to make a complete prosthesis.

### 3.2. Materials

In the conventional manufacturing process of a prosthesis, experts A. Creighton-Leroux, (CPO)c (May 2022) and J. Nokes, (CP)c (May 2022) stated that clear, heat-moldable thermoplastic polymer is used for initial diagnostic prosthesis due to its see-through nature which is very beneficial in locating the regions of the limb which need pressure relieving or reinforcing. However, these thermoplastic sockets might crack if they are used for too long. For the final definitive socket, layers of nyglass, fiberglass and carbon fiber are added to impart strength and durability in the prosthesis. However, alteration of the shape of this lamination is challenging after it is made. Furthermore, the entrapment of air bubbles in the thermoplastic or PVA (polyvinyl acetate) sheet occasionally mandates a remake.

C. Nagan (May 2022) pointed out the lack of empirical data about the structural strength of the materials used in 3D printing technologies and expressed concern over patients’ safety using a 3D-printed prosthesis. Many professionals such as L. Trevelin, (RTP)c (June 2022), D. Nelson, (CP)c (May 2022) and M. Rapaport, (CP)c (May 2022) were concerned about the strength of the materials used in 3D printing and the safety of patients using the devices made of those materials. Furthermore, the parts printed by FDM are not transparent because of inherent inter and intra-layer voids. Therefore, visual inspection to identify gaping for modifying the socket for adjustment becomes difficult. As expected, most of the experts also concluded that the surface quality of the traditionally manufactured device is superior when compared to the AM-processed part.

While discussing the manufacture of orthosis, the practitioners did not express any concern for the material properties of the materials owing to the type of use and functionality of the orthotic device.

Several research has been conducted and published testing different materials for 3D printing of O&P devices. A summary of the two processes is given in Table 1.

As for the prostheses, Branko et al. published a case study involving a patient with a missing thumb [35]. The researchers used a bio-based thermoplastic copolyester, Arnitel^®^ Eco, to 3D print the artificial thumb which was found to function sufficiently for the patient to hold, grab and pinch objects [35]. Leite et al. reported another case study involving a toddler’s prosthetic arm that was 3D printed using NinjaFlex™ material. The device was well received. The parents said that their child had not rejected or even expressed any concerns about using the device in his regular activities. The authors concluded that the device offered a high level of customisation at a significantly lower cost [36].

Regarding the application in orthoses, Brognara et al. compared the mechanical properties of standard PC (Polycarbonates) made orthosis with those made of ABS (Acrylonitrile Butadiene Styrene), PETG, PC and PLA using the FDM extrusion technology. The three-point bending test showed that standard and printed PC parts had similar strain and failure modes. The authors also compared the shape capture process by 3D scanning with traditional casting and reported 3D scanning to be an excellent alternative. They also developed a generative design (GD) workflow which can be used by the technicians to modify and customize designs as per customer requirements [37]. Sharma et al. examined the use of thermoplastic polyurethane (Ninjaflex and Filaflex polymer filaments) for orthotic foot insole by conducting tensile, bending and hardness tests on the printed part [38]. They concluded thermoplastic polyurethane exhibits superior properties in those tests.

### 3.3. Design and Fabrication Efficiency

During the interviews, experts such as C. Nagan (May 2022), S. Scott, (CP)c (May 2022), D. Moe, (CP)c (May 2022) and B. V. Lenthe, (CP)c (May 2022) mentioned that as the residual limb undergoes continuous volume changes in the initial days, patients need regular adjustments of the prostheses. Otherwise, patients would encounter several issues with the prostheses due to poor fit. If the prostheses do not fit properly with the residual limb, patients will experience pain and discomfort. Sometimes, pressure sores develop in some areas of the residual limb because of the non-uniform distribution of the load. Moreover, friction between the residual limb and socket results in irritation and skin problems often requiring a remake of the prosthetic device using a time-consuming and repetitive process.

While discussing the conventional manufacturing process, several experts such as A. Litner, (CPO)c (June 2022) and T. Chabot, (CP)c and (RTP)c (June 2022) pointed out that the manual manufacturing process is waste-producing and hazardous. Cutting and grinding the fiberglass and carbon fibers produces fine particles and dust, which are unsafe for health. In addition, the plaster of Paris and other cast materials which are used to fabricate the check socket for trial purposes are discarded which creates a large amount of waste. This wastage is not only an economic burden but also an environmental hazard.

To mitigate and alleviate those drawbacks, researchers developed methodologies to 3D print O&P devices. An overview of the two procedures is given in Table 1.

In regard to prostheses, Olsen et al. investigated the advantages and drawbacks of a fully digitized trans-radial diagnostic socket manufacturing process [39]. The researchers used an optical scanner to capture the shape, CAD software to modify the shape on a computer and FDM 3D printer to print a prosthetic device using PLA. They compared the prosthetic device made through the digital workflow with a prosthetic device processed traditionally. With the 3D-printed prosthetics, the patients were overall satisfied with the comfort level but suggested some adjustments to improve the suspension. Giri Ratnakar & Ramu focused on the creation of 3D models using computerized tomography (CT) scanning technology and the analysis of those models by finite element analysis [40]. The 3D model was extracted from the CT data using image processing tools. The obtained models were updated and converted into STL format during CAD modeling. In ANSYS, both static and dynamic conditions were tested on the designed prosthetic socket model, and necessary adjustments were made to the CAD model which was then 3D printed using ABS. The authors reported that the patients were satisfied with the 3D-printed transtibial prosthesis.

As for orthoses, Patel et al. used an FDM printer to print an AFO part using PLA in less than 6.5 h as opposed to days with conventional manufacturing processes. The authors opined that the use of additive manufacturing techniques had been demonstrated to expedite, streamline, and enhance the quality of customized items in the field of rehabilitation [41].

Li et al., in their study, created parametric models of orthosis using the Visual Programming Language in the CAD environment [42]. Five students from a nursing school participated in a brief training program (~15 min). They tested its feasibility in an orthosis design exercise and created five orthoses on their own in between eight and twenty minutes. The study demonstrated the viability of utilizing a parametric model for the design of 3D-printed orthoses and its improved usability for medical staff compared to the CAD technique. It also emphasized the advantages of using a parametric model over manual modeling training [42].

Farhan et al. compared the efficiency, accuracy, and dependability of 3D scanning with more conventional approaches for capturing foot and ankle morphology for the fabrication of orthoses. As per their findings, casting seemed to take longer than 3D scanning when compared to conventional procedures (2 to 11 min vs. 11 to 16 min). For both 3D scanning and conventional methods, inter-rater reliability (ICC 0.18–0.99) and intra-rater reliability (ICCs 0.25–0.99) were extremely diverse, with stronger agreement typically depending on the foot parameter evaluated [43].

### 3.4. Structural Strength

O&P professionals such as B. Pousett, (CP)c (May 2022), L. Janzé, (CP)c (May 2022), H. Gholizadeh, (CP)c (May 2022) and L. Schubert, (CP)c (May 2022) pointed out that the use of 3D printing for final “definitive” load-bearing lower-prostheses has been limited. Here, the term “load-bearing lower-prosthesis” means the prosthetic device which is worn at the lower part of the body, and which supports and carries the weight of the body. So far, the main applications of 3D printing have been restricted to the manufacture of cosmetic arms, components of upper arms and in some cases, the upper arm itself. In a few instances, diagnostic sockets have been produced in this method. Some experts pointed out that for the lower limbs, they prefer the traditional manufacturing techniques of thermoforming and lamination. For the upper limbs, in addition to the traditional methods, some businesses are already using existing 3D printing technologies, although on a limited scale. The main reason why clinicians still prefer traditional manufacturing techniques for O&P devices is the limited performance demonstrated by the O&P devices printed with commercial 3D printing technologies. Also, there have been no qualification standards developed yet to test the structural safety of 3D-printed O&P devices, which is hindering industrial adoption. The lower limb prostheses must withstand not only the body weight of the patients but also the loads that they carry in their professions.

Experts such as S. Scott, (CP)c (May 2022) and A. Litner, (CPO)c (June 2022) also expressed their concerns about the 3D printing technology and the 3D printed part. It was pointed out that lower limb prosthesis experiences a large amount of torque as the patient moves, which restricts the use of FDM technology for printing O&P devices. This is because of the inherent anisotropy of FDM parts resulting from the poor inter and intra-layer adhesion. Therefore, the AM technologies that impart nearly isotropic bulk material properties such as SLS offer a higher potential to print O&P devices.

As mentioned by some experts such as H. Gholizadeh, (CP)c (May 2022) clinicians rely on their experiences and typically do not perform any systematic tests to check the strength of the prosthetic parts that they manufacture. However, A. Lau, (CP)c (May 2022) mentioned that there is a standard to assess the structural strength of the prosthetic devices, ISO 10328:2016 Prosthetics—Structural testing of lower-limb prostheses—Requirements and test methods. Hence, it is of utmost importance that the 3D printed prosthetic devices are tested, as per ISO standards, to ensure that they qualify in terms of safety and strength which will eventually increase AM acceptance rate by the clinicians.

To assess the strength of the 3D-printed prosthetic sockets, Pousett et al. conducted an experiment which took around 9 h to print but required much less active time from a technician than traditional manufacturing methods [27]. Although both 3D printed and traditionally manufactured sockets passed the ultimate tensile strength test for static forces applied in gait, the 3D printed sockets failed at approximately half of the force of traditionally made sockets. Another issue raised during the expert interviews was that the 3D printed sockets break catastrophically, while traditionally made sockets would yield or tear slowly. Campbell et al. studied how the variation of infill percentage affects the 3D printed transtibial (TT) sockets [44]. They FDM printed nine TT sockets using PLA with 30%, 40% and 50% infill density and tested them for their ultimate strength as per ISO Standard 10328:2006. They found out that all of the nine sockets surpassed the 4480 N threshold set by the ISO Standard. Eight specimens failed at roughly twice the force threshold and one specimen failed at 5360 N [44]. It is to be noted that Both Pousett et al. and Campbell et al. used the AOPA Foot Project protocols to inform their methodologies—in addition to ISO 10328.

To evaluate the effects on orthoses, Banga et al. took feedback from patients who had reported heating and sweating of AFOs during long-term use [45]. The authors consulted with orthotists to create four AFOs of different designs in CAD software. Then they used finite element modeling and stress analysis to improve the designs. It was demonstrated by the biomechanics tests that prototype AFOs were either as good as or better than the standard AFO in their performances [45]. Lin et al., in their study, evaluated the strength of 3D-printed Anterior Ankle Foot Orthosis (AAFO) using the finite element (FE) approach [46]. The 3DP-AAFOs and traditional anterior AFO (TAAFOs) were designed with a 3.2 mm thickness. The experimental results were compared with results from the FEA model. The strength of the AAFO model was improved in the FE study by adding thickness at the neck of the AAFO design. Using 3DP-AAFO models, stress analysis was performed on a plantarflexion and a dorsiflexion moment. The stiffness of 3DP-AAFO was found to be 7.8 times higher than that of TAAFO. According to the FE results, increasing the 3DP-stiffness AAFO’s and reducing stress concentration might be accomplished by thickening the component up to 4.7 mm on the neck. The fracture might be avoided by increasing the necessary thickness around the neck of the 3DP-AAFO [46]. A summary of the two processes is presented in Table 1.

### 3.5. Functionality and Patient Satisfaction

Due to the lack of relevant study, the experts C. Ganzert, (CO)c (June 2022), J. Nokes, (CP)c (May 2022) T. Richardson, (CP)c (June 2022) and M. Pearce, (CP)c (June 2022) during their interviews expressed their concerns regarding the long-term functionality of 3D printed lower prostheses, which will bear the heavy load of the body. Because of that, 3D printing has been used for non-load-bearing devices such as orthoses and some upper-limb prostheses. Many practices across Canada and the world are now using 3D printing extensively for the manufacturing of orthoses.

Several research has been reported having assessed the functionality and patients’ feedback on the 3D printed O&P devices.

To evaluate the performance of additively manufactured prostheses, Cuellar et al. designed and printed a non-assembly hand prosthesis using PLA and tested the specimen for mechanical and functional performances [47]. The ultimate tensile strength was significantly higher than the required maximum load. As for the pinch force and the mechanical work, the non-assembly prosthetic hand required lower energy to close as compared to other body-powered prosthetic hands. The device was also assessed using the Box and Blocks Test (BBT) and the Southampton Hand Assessment Procedure (SHAP) test to assess the functionality. The test results showed a comparable level of functionality relating to other alternatives [47].

Copeland et al. reported that as high as 52% of users abandon upper limb prostheses due to the delay between amputation and prosthesis fitting [48]. They opined that fitting an amputee with a prosthesis within four weeks following the amputation would increase the likelihood of acceptance of the device. They opined that one way of reducing the high abandonment rate might be to utilize 3D printing for the rapid manufacture of the prosthetic device and get patients habituated to the use of prostheses as early as possible [48].

According to Abdelaal et al., producing a partial foot prosthesis (PFP) traditionally requires a lot of work and time [49]. In addition, the low regard for tarsal and mid-tarsal amputations has been attributed in part to the poor prosthesis fit that frequently results from conventional manufacturing techniques. As a result, both comfort and functionality may suffer greatly. Therefore, further improvement of current designs is required, along with the development of novel methods to make better-fitting and lighter prosthetic devices. Since medical-grade silicones cannot be printed by commercial 3D printers, indirect use of AM technique can be realized by printing a positive mold using 3D printed materials and then casting medical-grade silicone into 3D printed molds [49].

As for the orthoses, Geoffroy et al. described a brand-new cranial remodeling orthosis (CRO) helmet design that was created using 3D scanning and printing technology to treat infantile plagiocephaly [50]. To define the design and the manufacturing process utilizing a topological optimization method, the project identified the current limitations and developed design requirements and acceptance criteria. The ABS material utilized in 3D printing had limitations, and several improvements to the process were suggested. The primary innovation was the creation of a 3D scanning and 3D printing technology to rectify infantile plagiocephaly and produce a CRO helmet that satisfied use and production requirements while offering an appropriate organic shape [50].

Vasiliauskaite et al. investigated the possibility of reducing excessive plantarflexion with drop foot gait by additively manufacturing an AFO with predefined ankle stiffness [51]. For that purpose, they determined the optimal stiffness using hinged AFO and then digitally designed and additively manufactured a leaf-spring type AFO using polyamide-12 powder with desired stiffness. They reported that although the additively manufactured leaf-spring type AFO decreased the excessive plantarflexion problem, the impact was moderately inferior as compared to the hinge AFO [51]. Both processes are summarized in Table 1.

## 4. Barriers to Adoption of AM in the Canadian Context

Having compared the insights from both the experts from the interviews and the published literature in five key areas, we can opine that 3D printing provides an overwhelming advantage for the manufacture of non-load-bearing devices. As a result, many businesses are now adopting 3D printing for the manufacture of orthosis and non-load-bearing prostheses. 3D printing also provides a greater advantage in lowering the manufacturing cost of prosthetic devices and significantly improving the efficiency of the design and fabrication processes. As the AM techniques substitute the conventional process with 3D scanners, 3D printers and digital environment, it eliminates many manual steps, thus making the execution and iteration of the design and fabrication process much more time-efficient and cost-effective.

While discussing the aforementioned advantages of additive manufacturing, some practitioners pointed out that although they were aware of those benefits, one key factor that might preclude them from acquiring advanced 3D printing machines was the cost-benefit analysis of the procurement. A. Litner, (CPO)c (June 2022) stated that, as many O&P practices in Canada are small and medium-sized facilities, it might not be economically viable for them to purchase expensive machines given their current level of turnover. However, L. Schubert, (CP)c (May 2022) stated that many such practices have begun to adopt 3D printing for some of their products by outsourcing the fabrication to large manufacturing companies such as Additive America. On the other hand, J. Nokes, (CP)c (May 2022) informed us that large companies such as PBO Group (Canada) have started utilizing 3D printing by purchasing machines and hiring dedicated technicians to operate those machines.

However, experts also pointed out some limitations of the 3D-printed prosthetic parts in terms of the limited material choice and structural strength of the printed parts. The materials which are used in the lamination of the final socket, such as nyglass, carbon fiber and fiber glass, are not economically available for 3D printing. Moreover, the structural strength and durability of the 3D printed load-bearing parts have not been systematically and rigorously verified yet in literatures or via an appropriate ISO standard. These limitations severely restrict the mass adoption of 3D printing techniques for the manufacture of a lower-limb prosthesis. Findings from the published articles on materials and structural strength vary depending on the printer type, the material used, part type, experimental set-up, and other parameters. Some articles report an abrupt failure of the 3D-printed parts when subjected to tests, whereas others state better results as compared to conventional ones.

The supposed weakness in the strength of the 3D printed parts arises from the anisotropic printing direction of the material extrusion i.e., the FDM process. This limitation can be overcome by choosing a layer-stacking scheme or by changing the print direction in such a way that the load is applied across the layer [52]. In many cases, FDM does not provide any smooth surface finish. To rectify that issue, sanding and post-processing can be done to increase the surface's smoothness. In addition, other alternative 3D printing processes such as selective laser sintering (SLS), material jetting (MJ) and vat photopolymerization (VPP) methods can also be used which are capable of providing isotropic strength to the material parts. However, as discussed above, those machines may not be economically viable for small and medium-sized facilities. Moreover, restrictions on the size of the prosthetics due to the build volume of the machines, and the human skin compatibility of the materials used in those machines should also be considered.

## 5. Conclusions

The purpose of this paper is to review the industry outlook of additive manufacturing for O&P devices by linking the perspectives of the O&P professionals with the published research. The insights gathered have been categorized into five key areas: cost, material, design and fabrication efficiency, the structural strength of the parts, and functionality and patient satisfaction. Important findings are:

- The cost of the 3D-printed prosthetic device was reported to be approximately 56–95% lower than that of the laminated device.
- The reported reduction of fabrication cost is mainly for the printing of the socket. Other components are still needed to be connected to the socket to make a complete prosthesis.
- Nyglass, fiberglass and carbon fiber are added to the final socket in the conventional method. Practitioners opined that 3D printing would not be able to replicate the properties of those materials.
- Orthotic devices made of ABS, PETG, PC and PLA using the FDM extrusion technology were reported to have the same characteristics as traditional PC-made devices.
- The fabrication methods for the O&P devices in the conventional method are wasteful and hazardous. The design and repetition are time-consuming.
- 3D printing has been reported to significantly improve design and fabrication efficiency.
- Practitioners were concerned about the structural strength of the FDM-printed lower-limb prostheses.
- Published articles also reported unsatisfactory results for the strengths of the lower-limb prostheses. However, acceptable results were reported for orthotic devices.
- Regarding functionality and patient satisfaction, published articles report better or similar functionality and patient satisfaction for the 3D printed upper-limb prosthesis, which has been usually agreed upon by many experts.
- However, there has been a lack of comparable data to reach any meaningful conclusion for the lower-limb prosthesis.

There is a huge potential for researchers in the field to examine 3D-printed O&P devices in terms of materials, structural strength, and other key areas. Researchers should also focus on developing rapid qualification strategies to help the industry adopt 3D printing. Ensuring and demonstrating the safety and quality of the printed parts is of paramount importance and is a strong requirement for a wide-scale technology transition.

## Figures and Tables

**Table 1:** Comparison between Conventional and Additive Manufacturing for O&P devices.

Key Fields	Conventional Manufacturing	Additive Manufacturing
Overall Manufacturing Cost	Conventionally manufactured O&P devices cost higher than that manufactured by AM methods. The higher cost is due to the labour-intensive, and wasteful nature of the conventional manufacturing process.	Published articles report a reduction in fabrication cost for the O&P devices. Although, as for prosthetics, other components are to be assembled with the socket to make a complete device.
Materials	Transparent polymer is used for the diagnostic prosthesis, and layers of nyglass, fiberglass and carbon fiber are added for the final device.	FDM cannot print transparent or the materials for the final prosthetic device. However, orthotic devices made of ABS, PETG, PC and PLA using the FDM have been successfully tested.
Design and Fabrication Efficiency	The traditional method for O&P devices is wasteful, time-consuming, and hazardous.	Due to its inherent nature, 3D printing generates minimal wastage and improves efficiency.
Structural Strength	The conventional manufacturing method imparts sufficient strength to the O&P devices.	FDM printed devices exhibit anisotropic strength i.e., they are weak along the printing direction.
Functionality and Patient Satisfaction	Conventionally manufactured O&P devices provide proven functionality and satisfaction to patients.	3D-printed orthotic and upper limb prosthetic devices provide superior or comparable functionality and patient satisfaction. As for the lower-limb prosthesis, the findings are inconclusive.

## Data Availability

Data presented in this study are available on request from the corresponding author.

## Supplementary Materials

The following supporting information can be downloaded at: https://www.mdpi.com/article/10.3390/polym15061506/s1, Section S1: Table S1: Interview List of O&P Professionals, and Section S2: Interview Questionnaire.
