# Supplementary material for: Polymer-Based Additive Manufacturing for Orthotic and Prosthetic Devices: Industry Outlook in Canada
Source: Polymers (Basel). 2023 Mar 17;15(6):1506. doi: 10.3390/polym15061506 (PMC10057521; doi:10.3390/polym15061506)
Supplement: Supplementary file 1 [file polymers-15-01506-s001.zip › polymers-2240022-supplementary.pdf]

Supplementary Materials

Section S1: List of O&P Professionals Interviewed

Table S1. Interview list of O&P Professionals.

Serial	Name	Organization	Interview Date (CST)
1	Adriel Lau, (CP)c	BC Prosthetic & Orthotic Services (Kelowna, BC)	5/26/2022 8:00 AM
2	Andrew Litner, (CPO)c	PBO Group (St. Catharines, ON)	6/6/2022 10:30 AM
3	Angela Creighton-Leroux, (CPO)c	Kelowna General Hospital (Kelowna, BC)	5/10/2022 1:00 PM
4	Bradley Van Lenthe, (CP)c	Freedom Prosthetics (Woodbridge, ON)	5/9/2022 11:30 AM
5	Brittany Pousett, (CP)c	Barber Prosthetics Clinic Inc. (Vancouver, BC)	5/12/2022 12:30 PM
6	Calvin Ngan, Researcher, PhD Candidate, University of Toronto	Holland Bloorview Kids Rehab Hospital (Toronto, ON)	5/24/2022 1:00 PM
7	Carl Ganzert, (CO)c	Hodgson Orthopedic Group (Surrey, BC)	6/7/2022 2:00 PM
8	Christina Fung, (CP)c	Sunnybrook Centre for Independent Living (Toronto, ON)	5/19/2022 11:30 AM
9	David Moe, (CP)c	Barber Prosthetics Clinic Inc. (Vancouver, BC)	5/12/2022 3:00 PM
10	Duane Nelson, (CP)c	Synergy Prosthetic Services Inc (Edmonton, AB)	5/27/2022 9:00 AM
11	Hossein Gholizadeh, (CP)c	Ampos Orthopaedics (Ottawa, ON)	5/24/2022 11:00 AM
12	Jenna Barnert, (CP)c	Cascade Prosthetic Services Ltd. (Calgary, AB)	5/27/2022 3:00 PM
13	Jeremy Nokes, (CP)c	PBO Group (St. Catharines, ON)	5/17/2022 7:00 AM
14	Laura Trevelin, (RTP)c	Nova Scotia Rehabilitation and Arthritis Center (Halifax, NS)	6/8/2022 9:30 AM
15	Loren Schubert, (CP)c Head of O&P Program at BCIT	British Columbia Institute of Technology (Burnaby, BC), Hodgson Orthopedic Group (Surrey, BC)	5/26/2022 3:30 PM
16	Louis Janzé, (CP)c	Fraser Valley Prosthetics Ltd. (Abbotsford, BC)	5/19/2022 4:00 PM
17	Malena Rapaport, (CP)c	Barber Prosthetics Clinic Inc. (Vancouver, BC)	5/20/2022 10:30 AM
18	Michael Pearce, (CP)c	Ottobock Healthcare Canada Ltd (Burlington, ON)	6/15/2022 6:30 AM
19	Michael Prystai, (CPO)c	Vancouver Prosthetics & Orthotics Inc. (Vancouver, BC)	5/12/2022 11:30 AM
20	Steve Scott, (CP)c	Cascade Prosthetic Services Ltd. (Calgary, AB)	5/10/2022 1:15 PM
21	Tania Chabot (Gripper), (CP)c and (RTP)c	Russell Prosthetics Ltd. (New Westminster, BC)	6/2/2022 9:30 AM
22	Tessa Richardson, (CP)c	Barber Prosthetics Clinic Inc. (Vancouver, BC)	5/13/2022 9:00 AM

Section S2: Interview Questionnaire

▪ *Preliminary Profile Questions*

- Can you state your name please?
- What is the name of your company?
- How many years have you worked in this industry?

▪ *Job/Position*

- What are your roles and responsibilities ?

▪ *Identification and determination of the Severity of the Problem*

- Which process/technique is used in your facility for manufacturing prosthesis or orthosis (hand casting/ digital scanning or any other method)?
- What materials are used for the manufacture of diagnostic socket?
- What materials are used for the manufacture of definitive socket?
- What are the problems you are having while manufacturing prosthesis or orthosis?
- What issues do the patients face with the prosthesis or orthosis that are made using the current process?
- If you have problems with your current manufacturing process, how often would you say these problems occur? Do these problems cost your business time or money?
- Have you researched other types of technology regarding manufacturing of prosthesis or orthosis? Have you tried 3D printing so far?
- If you are familiar with the 3D printed prosthesis or orthosis, what is your opinion on that? (like concerns, issues, advantages etc)

▪ *Channels*

- Who are the current suppliers of prosthetics materials, components and accessories?

▪ *Revenue Streams*

- What is the usual range for the cost of the upper limb prostheses?
- What is the usual range for the cost of the lower limb prostheses?
- How often do you upgrade your technology?

▪ *Customer Relationships*

- When was the last time you purchased/integrated a product/technology for manufacturing prosthesis or orthosis?

▪ *Success metrics.*

- What do/did you consider (i.e. features) when looking for manufacturing the best prosthesis or orthosis?
